# Determination of liver fibrosis stages in Egyptian chronic hepatitis B patients by a noninvasive tool

**DOI:** 10.3906/sag-1812-165

**Published:** 2019-08-08

**Authors:** Waleed Mohamed SERAG, Magdy Mahmoud MOHAMED, Basem Eysa ELSAYED, Sara Mahmoud ABD-ELHAMED

**Affiliations:** 1 Department of Chemistry, Faculty of Science, Suez University, Suez Egypt; 2 Department of Biochemistry, Faculty of Science, Ain Shams University, Cairo Egypt; 3 National Hepatology and Tropical Medicine Research Institute, Cairo Egypt; 4 Chemical Administration, Suez Egypt

**Keywords:** HBsAg, stage of fibrosis, angiopoietin-like protein 2, APRI, FIB-4

## Abstract

**Background/aim:**

Hepatitis B virus (HBV) infection is the leading cause of liver fibrosis (LF). The prognosis and management of patients with chronic hepatitis B virus depend on the amount and progression of liver fibrosis. Angiopoietin-like protein 2 (Angptl2) is not only a chronic inflammatory mediator, but also a tissue-remodeling factor. The aim of this study is to explore the predictive value of Angptl2 in different fibrosis stages in patients chronically infected with HBV.

**Materials and methods:**

Eighty patients with chronic HBV infection undergoing Fibroscan were included. Serum concentrations of Angptl2 were detected using a commercial ELISA kit.

**Results:**

Angptl2 levels were significantly associated with liver fibrosis stages (P = 0.02). The area under the curve (AUC) of Angptl2 for distinguishing patients who showed significant fibrosis (F2–F4) was****70.2%. Angptl2 with fibrosis-4 (FIB-4) and Angptl2 with AST/platelets ratio (APRI) performed best with an AUC of 92.5%.

**Conclusion:**

In patients with chronic HBV infection, Angptl2 level represents a potential biomarker independently associated with fibrosis stages. The combination of Angptl2 with FIB-4 or Angptl2 with APRI performed better than the existing models for diagnosing significant fibrosis.

## 1. Introduction

Chronic hepatitis B (CHB) is defined as chronic necroinflammatory disease. It is the 10th leading cause of death in the world [1]. Fibrosis is a dynamic process characterized by excessive accumulation of extracellular matrix (ECM) proteins, resulting in pathological collagen deposition [2]. Hepatic fibrogenesis begins with the stimulation of inflammatory immune cells to secrete cytokines [3]. Typical wound healing for chronic liver diseases is defined by an increase in the expression of some cytokines, growth factors, and metalloproteinases with proangiogenic action [4]. In advanced stages, the liver contains approximately 6 times more ECM than normal [5]. Liver fibrosis is a middle stage in the course of chronic HBV infection, which will develop into cirrhosis and eventually hepatocellular carcinoma (HCC) if not treated at the early stage. The risk of developing cirrhosis depends on the degree or stage of fibrosis in the liver [6]. Fibroscan is a noninvasive test that replaces liver biopsy [7]. Liver biopsy merely offers a snapshot image of the underlying histology at a given time point [8]. Biomarkers of fibrosis are commonly divided into two main categories: direct and indirect markers. Direct and indirect markers can be used alone or more frequently in combination to increase the diagnostic reliability [9,10]. Cytokines and regulatory molecules are essential mediators in the host’s adaptive immune response to HBV and viral clearance. Imbalance in cytokine production plays a key role in the development of liver damage, necroinflammation, and subsequent fibrosis [11]. However, only a few studies have addressed the diagnostic accuracy of fibrosis-associated cytokines [12]. Angiopoietin-like protein 2 (Angptl2) is a glycoprotein [13] belonging to the angiopoietin-like family [14]. It is secreted by hepatocytes [15]. Excess Angptl2 signaling causes chronic inflammation and subsequently pathological irreversible tissue remodeling [16]. The systemic inflammation is the obvious link between an increase in circulating levels of Angptl2 and chronic diseases. In chronic low-grade inflammation, sustained overproduction of proinflammatory Angptl2 could contribute [17]. The regulatory X protein of hepatitis B (HBx) activates the transcription factors nuclear factor kappa beta (NF-κB), which promotes HBV replication [18,19]. Angptl2 also activates the integrin α5β1/Rac1/NFκB-dependent pathway, subsequently resulting in transcription of several proinflammatory cytokines [20,21]. Angptl2 signaling through integrin a5b1 increases tissue inflammation and ECM remodeling, resulting in activation of tissue remodeling [16,20]. It contributes to the acceleration of tissue fibrosis through activation of the transforming growth factor beta (TGF-β1)/Smad signaling pathway, like in renal fibrosis [22]. High Angptl2 concentrations are a marker of cellular dysfunction, associated with systemic inflammation [20]. The aim of this study is to explore the predictive value of Angptl2 in different fibrosis stages in patients chronically infected with HBV.

## 2. Materials and methods

This study population consisted of ten control volunteers and eighty patients with CHB infection of different fibrosis stages from the National Hepatology and Tropical Medicine Research Institute. HBV patients were diagnosed based on positive HBV surface antigen (HBsAg) and fluctuated alanine aminotransferase (ALT) and HBV-DNA (≥2000 IU/mL). Full clinical, biochemical, and hematological data were recorded for all patients. The patients were personally asked about the time of their infection, whether they were being treated or not, and the type of treatment prescribed for them. Liver stiffness was measured by Fibroscan. HBV cases were divided into fibrosis stages from F0 to F4. Noninvasive markers of fibrosis, including APRI, AST/ALT ratio (AAR), and FIB-4 score were calculated. Serum Angptl2 concentrations were determined using the Human Angptl2 Assay Kit according to the manufacturer’s instructions. Patients with any clinically significant diseases were excluded. The full, detailed clinical trial protocol was registered under serial number 7-2016. A 3-mL sample of peripheral blood was taken from both patients and controls. All blood samples were centrifuged and stored at –20 °C for the measurement of serum Angptl2 levels. One-way analysis of variance was used for comparison of multiple groups. Differences of normally and nonnormally distributed variables between the groups were analyzed using Student’s t-test. To assess differences in proportions, the chi-square test was used. The predictive performance expressed as areas under the receiver-operating characteristic curves (AUC ROCs), sensitivity, specificity, positive predictive value (PPV), and negative predictive value (NPV). All data were expressed as the mean ± standard deviation (SD) or proportions [23]. 

## 3. Results

The control group included ten volunteers including 8 females and 2 males, their ages ranging from 15 to 20 years old. We divided the HBV patients into three groups: Group 1 (F0–F1), “no/mild hepatic fibrosis”, included 33 patients (41.25%), 25 of whom were male and 8 female with mean age of 39.36 ± 11.56 years. Group 2 (F2), “significant hepatic fibrosis”, included 9 patients (11.25%), with 8 males and 1 female with mean age of 37.44 ± 7.7 years. Group 3 (F3–F4), “severe hepatic fibrosis and cirrhosis”, included 38 patients (47.5%), 28 of whom were males and 10 female with mean age of 49.42 ± 10.32 years. The mean age of all patients was 43.93 ± 11.74 years. In this study population, 61 (76.25%) were male and 19 (23.75%) were female. In the control group, serum Angptl2 concentration was 0.93 ± 0.47 ng/mL, which was different from that in patients with no/moderate fibrosis, so early fibrosis stages could be distinguished. With progression in the fibrosis stages, the Angptl2 concentrations were significantly increased (P = 0.002), as seen in Table 1. Serum Angptl2 concentrations were not just positively associated; they were also significantly correlated with liver stiffness measurements by Fibroscan (r = 0.249, P = 0.026). We compared Angptl2 concentrations with the commonly known noninvasive fibrosis markers (APRI, FIB-4, and AAR). Overall, there was a positive association between Angptl2 and APRI, FIB-4, and AAR in the three patient groups of fibrosis, as seen in Table 1.

**Table 1:** Comparison between noninvasive markers at different fibrosis stages.

P-value	F3–F4 (n = 38)	F2 (n = 9)	F0–F1 (n = 33)	Parameters
0.003*	1.3 ± 0.58	0.95 ± 0.27	0.94 ± 0.31	AAR
<0.001*	1.21 ± 1.06	0.80 ± 0.90	0.31 ± 0.1	APRI
<0.001*	4.13 ± 4.60	1.65 ± 1.80	0.92 ± 0.53	FIB-4
<0.001*	24.41 ± 17.68	7.77 ± 0.32	5.02 ± 1.03	Fibroscan (kPa)
0.020*	3.02 ± 1.65	2.77 ± 2.71	1.79 ± 1.76	Angptl2 (ng/mL)

Significant correlations were found between Angptl2 and age, AAR, and Fibroscan (r = 0.359, P = 0.001; r = 0.242, P = 0.031; r = 0.249, P = 0.026, respectively). Angptl2 showed areas under the receiver operating characteristic curve of 0.702 for distinguishing patients with significant fibrosis (F2–F4) with a sensitivity of 87.23% and NPV of 71.4% by using a cut-off value of >1.1. AUC ROCs of the three biomarkers at different cut-offs for the diagnosis of F2–F4 fibrosis were calculated. Angptl2 is superior to AAR with AUCs of 70.2% and 67.8% for predicting significant fibrosis (F ≥ 2), respectively, as presented in Table 1 and the Figure.

**Figure:**
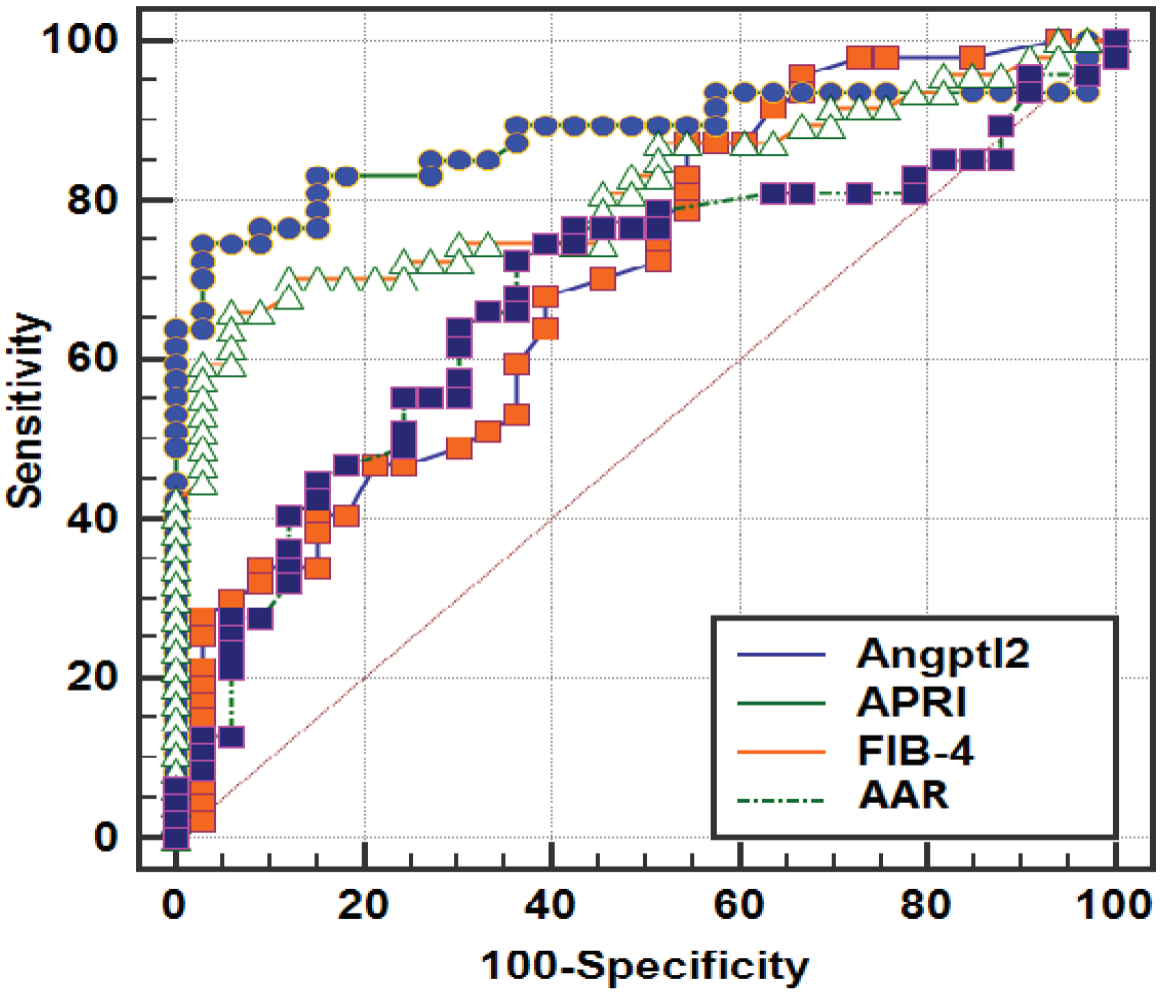
AUC of Angptl2, AST (aspartate aminotransferase) and platelet ratio index (APRI), FIB-4 index, and AST/ALT ratio to distinguish patients with significant fibrosis among the HBV patients.

When Angptl2 was combined with APRI, FIB-4, and AAR for the assessment of significant fibrosis, AUC ROCs were enhanced significantly (P < 0.001; Table 2).

**Table 2:** Diagnostic performance of noninvasive models predicting significant liver fibrosis.

Index	AUC	Sensitivity (%)	Specificity (%)	PPV (%)	NPV (%)	Cut-off
Angptl2	70.2%	87.23	45.45	69.5	71.4	>1.1
AAR	67.8%	72.34	63.64	73.9	61.8	>0.9
APRI	87.8%	74.47	96.97	97.2	72.7	>0.489
FIB-4	81.2%	65.96	93.94	93.9	66.0	>1.58
Combination of Angptl2 and AAR	88.75	95.74	78.79	86.54	92.86	
Combination of Angptl2 and APRI	92.5	87.23	100	100	84.61	
Combination of Angptl2 and FIB-4	92.5	93.62	90.91	93.62	90.91	

We observed that serum levels of Angptl2 were higher in patients who were not treated, (40%) with a mean value of 3.08 ng/mL, compared to those receiving treatment (60%), with a mean value of 2.1 ng/mL. We compared Angptl2 concentrations with log10 (HBV-DNA) and ALT levels in all HBV patients. Angptl2 levels increased in the untreated group compared to the treated group and both log10 (HBV-DNA) and ALT levels decreased. Log10 HBV-DNA levels were 7.07 and 4.19, while ALT levels were 35.51 and 29.31 in the treated group and the untreated group, respectively. In all patients, we compared the Angptl2 levels in the patients infected more than 1 year ago (71.25%) and the recently infected patients (<1 year) (28.75%). We found higher Angptl2 levels for older infections (>1 year), with a value of 2.81 ng/mL, than those for recent infections (<1 year), with a value of 1.61 ng/mL. We observed Angptl2 levels in HBV patients with normal levels of ALT who were not treated. In our study, we found that despite the normal levels of ALT, the Angptl2 serum levels were greater with higher liver fibrosis stages. For F0–F1, the level was 2.16 ng/mL; for F2, 4.53 ng/mL; but for F3–F4, 3.08 ng/mL. Thirty-nine (48.74%) of the HBV patients in our study had ultrasounds. We found that in the patients in the F0–F1 group, with bright liver, the mean Angptl2 value was 1.88 ng/mL. Patients in the F2 group, with chronic diffuse parenchymatous liver disease, had an Angptl2 value of 2.5 ng/mL. For the patients in the F3–F4 group, with splenomegaly and cirrhotic liver, the mean Angptl2 value was 3.15 ng/mL.

## 4. Discussion

Although liver stiffness measurement (LSM) was positively correlated with liver fibrosis stage [24], serum ALT levels >2  times the  upper limit of normal (ULN) have reduced the accuracy of transient elastography in detecting the early stages of fibrosis in CHB [25]. TE is only recommended for CHB patients with normal or elevated ALT not exceeding 5-fold the ULN [26]. Angptl2 affects liver fibrosis in HBV patients [20]. Angptl2 mRNA is expressed in the liver and secreted by hepatocytes. Additionally, high levels of Angptl2 protein were positively correlated with liver cirrhosis in HCC patients [15]. Angptl2 is a central regulator of multiple inflammatory mediators [27], including interleukin-6 (IL-6) and TGF-β1, found to lead to activation of collagen expression and consequentially affect tissue repair and fibrosis [28]. The HBx of HBV was found to stimulate angiogenesis directly [29]. Angptl2 maintains tissue homeostasis by inducing angiogenesis [30]. With all this evidence, it seems reasonable to assume that Angptl2 plays a significant role in liver fibrosis in HBV patients. Therefore, in this study including 80 HBV patients, it was demonstrated that serum Angptl2 concentrations are significantly correlated with LSM by Fibroscan. We found a significant difference in the Angptl2 levels between F0–F1 and F3–F4, which allowed us to differentiate severe fibrosis from mild fibrosis. This is an advantage to Angptl2, as LSM results could be considered mainly false negatives because of macronodular cirrhosis, which is more common in chronic hepatitis B [31]. We found a positive correlation between Angptl2 in different fibrosis stages and APRI and FIB-4, and a significant correlation between Angptl2 and AAR. Detection of significant fibrosis (F ≥ 2) is the most important clinically relevant endpoint in patients with CHB, which indicates that patients need antiviral treatment [23]. A cut-off of Angptl2 value of >1.1 was chosen, and the AUC of Angptl2 was 70.2%. Our results are higher compared to a previous study [11] that showed AUCs of APRI and FIB-4 of 0.66 and 0.63. Angptl2 was higher when it was compared to another study, which showed AUC ROCs of Fib-4 (0.70348), APRI (0.68627), and AAR (0.57636) for significant fibrosis [26]. Currently, Angptl2 is superior to AAR for the detection of significant fibrosis, with AUCs of 70.1 and 67.8, respectively. In order to increase the diagnostic accuracy of noninvasive tests, combined models utilizing two or more tests have been applied for replacing liver histology [32]. When Angptl2 was combined with APRI, FIB-4, and AAR for assessment of significant fibrosis, AUC ROCs were enhanced significantly. The AUC ROC of the combination of Angptl2 and FIB-4 or Angptl2 and APRI for predicting significant fibrosis was 0.92, significantly superior to APRI, FIB-4, and AAR. Starting treatment for HBV patients is a critical step. The treatment is started only when the HBV DNA levels are above 2000 IU/mL, ALT is >2 ULN, and active hepatic inflammation or fibrosis is seen [33]. We observed that Angptl2 in the 3 different fibrosis groups was always higher in the untreated group than the treated group. By contrast, both log10 (HBV-DNA) and ALT levels were decreased in the untreated group in comparison with the treated group. Consequently, Angptl2 proved to be useful to monitor the effectiveness of HBV treatment and for use as an indicator to start treatment. Significant fibrosis is not rare in CHB patients with normal ALT [34], so we measured Angptl2 levels in HBV patients with normal levels of ALT who were not treated. We found that despite normal ALT levels, the Angptl2 concentrations were greater with hepatic fibrosis progression. In the three different fibrosis stages, Angptl2 levels were found higher in old infections (>1 year) than recent infections (< 1 year), which can be an indicator of the inflammation process, since chronic HBV infection is a dynamic condition in which inflammatory response via antiviral T cells arises [35]. Angptl2 proved that it can be used successfully as an indicator of liver fibrosis in many ways.

Angptl2 concentration levels increased with the progression of fibrosis stages, significantly so in cases of significant fibrosis in patients with CHB infection. The combination of Angptl2 and FIB-4 or Angptl2 and APRI offers an easy possibility to diagnose significant fibrosis and its performance is superior to that of existing scores of APRI, FIB-4, and AAR.

## Acknowledgment

We acknowledge all the physicians of the National Hepatology and Tropical Medicine Research Institute for their help in samples collection and study.

## Ethical consideration

Written informed consent was obtained from all subjects. Each subject had the right to accept or reject participation. Confidentiality of collected data was ensured. The study was approved by the ethics committee of the National Hepatology and Tropical Medicine Research Institute and registered under serial number 7-2016. The study was organized and conducted according to the Declaration of Helsinki for human subject research.
